# A socioecological framework for research on work and obesity in diverse urban transit operators based on gender, race, and ethnicity

**DOI:** 10.1186/s40557-017-0171-2

**Published:** 2017-05-17

**Authors:** BongKyoo Choi, Peter Schnall, Marnie Dobson, Haiou Yang, Dean Baker, YoungJu Seo

**Affiliations:** 1 Center for Occupational and Environmental Health, University of California Irvine, 100 Theory, Suite 100, Irvine, CA 92617 USA; 2 Department of Sociology, University of Massachusetts Amherst, 200 Hicks Way, Amherst, MA 01003 USA

**Keywords:** Los Angeles, Minority, Work stress, Health behaviors, Weight gain, Injuries, Disparities

## Abstract

Urban transit (bus and rail) operators, totaling nearly 700,000 persons, are one of the heaviest occupational groups in the United States (US). Little is known about occupational risk factors for weight gain and obesity and their interrelationship with health-related behaviors, particularly among female minority (African Americans and Hispanics) transit operators who are at greater risk for obesity. As a step towards developing successful obesity interventions among urban transit operators, this paper aims to present a new socioecological framework for studying working conditions, chronic strain, health-related behaviors, weight gain/obesity, and obesity disparity in diverse urban transit operators based on gender, race, and ethnicity. Our framework is a synthesis of several different theories and disciplines: the resource-work load model (work stress), occupational ergonomics, the theory of intersectionality, and worksite health promotion. The framework was developed utilizing an extensive literature review, results from our on-going research on obesity, input from focus groups conducted with Los Angeles transit operators as well as interviews and meetings with transit operator stakeholders (management, unions, and worksite transit wellness program), and ride-along observations. Our hypotheses highlighted in the framework (see Fig. 1) are that adverse working conditions, largely characterized as a combination of high demands and low resources, will increase the risk for weight gain/obesity among transit operators directly through chronic strain and hypothalamic dysfunction (hyper-and hypo-activations), and indirectly through health-related behaviors and injuries/chronic severe pain. We also hypothesize that the observed increase in adiposity among female minority operators is due to their greater exposure to adverse occupational and non-occupational conditions that reflect their intersecting social identities of lower social class and being a minority woman in the US. Our proposed framework could greatly facilitate future transit worksite obesity studies by clarifying the complex and important roles of adverse working conditions in the etiology of weight gain/obesity and obesity disparity among transit operators and other working populations.

## Background

There are about 5.7 million commercial motor vehicle operators, including 0.7 million bus and urban transit operators in the United States (US) [1–3]. Obesity (>30 kg/m^2^ of body mass index (BMI)), an excessive accumulation of body fat, is a key health issue among US urban transit operators (bus and rail operators) [4–9]. Urban transit operators (of whom 50% are minorities) [1] were ranked first and second in obesity prevalence among 41 US male and female occupational groups, respectively [4]. In addition, about 20–33% of transit operators are minority women [1, 5] who have a much higher prevalence of obesity compared to male and other female operators (52–65% vs. 37–41%) [9]. Urban transit operators also have a high risk of hypertension, metabolic syndrome, cardiovascular disease (CVD), Type II diabetes mellitus, depression, non-fatal occupational injuries, chronic back and neck pain, and sickness absence [3, 7, 8, 10–21].

Despite wide-spread recognition that obesity is a problem among transit operators, little is known about the occupational risk factors for weight gain and obesity in urban transit operators or the reasons for the much greater obesity risk in minority women (African Americans and Hispanics) transit operators [9, 22]. In addition, to the best of our knowledge, no successful transit worksite obesity intervention studies exist [1, 5]. A search of the literature reveals two efforts: French et al. [5] conducted an 18-month worksite obesity intervention study mainly targeting increased availability of healthy vending machine choices at division buildings in transit operators in Minneapolis, USA. However, the intervention study did not successfully prevent or reduce obesity among transit operators. Another 18-month intervention study was carried out by Hedberg et al [23] for improving CVD risk factors among a group of German workers, including bus drivers. The contents of the interventions were health education and group or individual activities on exercise, diet, and stress coping. However, the level of BMI increased over the intervention period in both the intervention and control groups and no intervention effect on BMI was observed in the German study. Neither study addressed structural aspects of driving among transit operators.

One critical barrier to progress in worksite obesity studies among US transit operators is the lack of a good theoretical framework to guide research on work and weight gain/obesity. For example, the theoretical framework on work and health in transit operators by Ragland et al. [24] is too general to be used for research on work and obesity, although it suggests that high job demands and low job resources can increase the risk for disease and injury among transit operators through health-related behaviors or stress. No specific information about job demands or resources of transit operators as risk factors for obesity is available in their framework. Also, like most contemporary etiological frameworks on work and obesity in working populations [25] or of single occupational groups (e.g., professional firefighters) [26], their framework cannot explain appropriately the disparities in obesity by gender and race/ethnicity among US transit operators. Moreover, mechanistically, there is also no consideration in available frameworks [24–26] of the role of injuries/chronic severe pain that are prevalent among transit operators [16, 17, 19, 27] and which may function as a possible pathway from adverse working condition to weight gain/obesity.

**Fig. 1:**
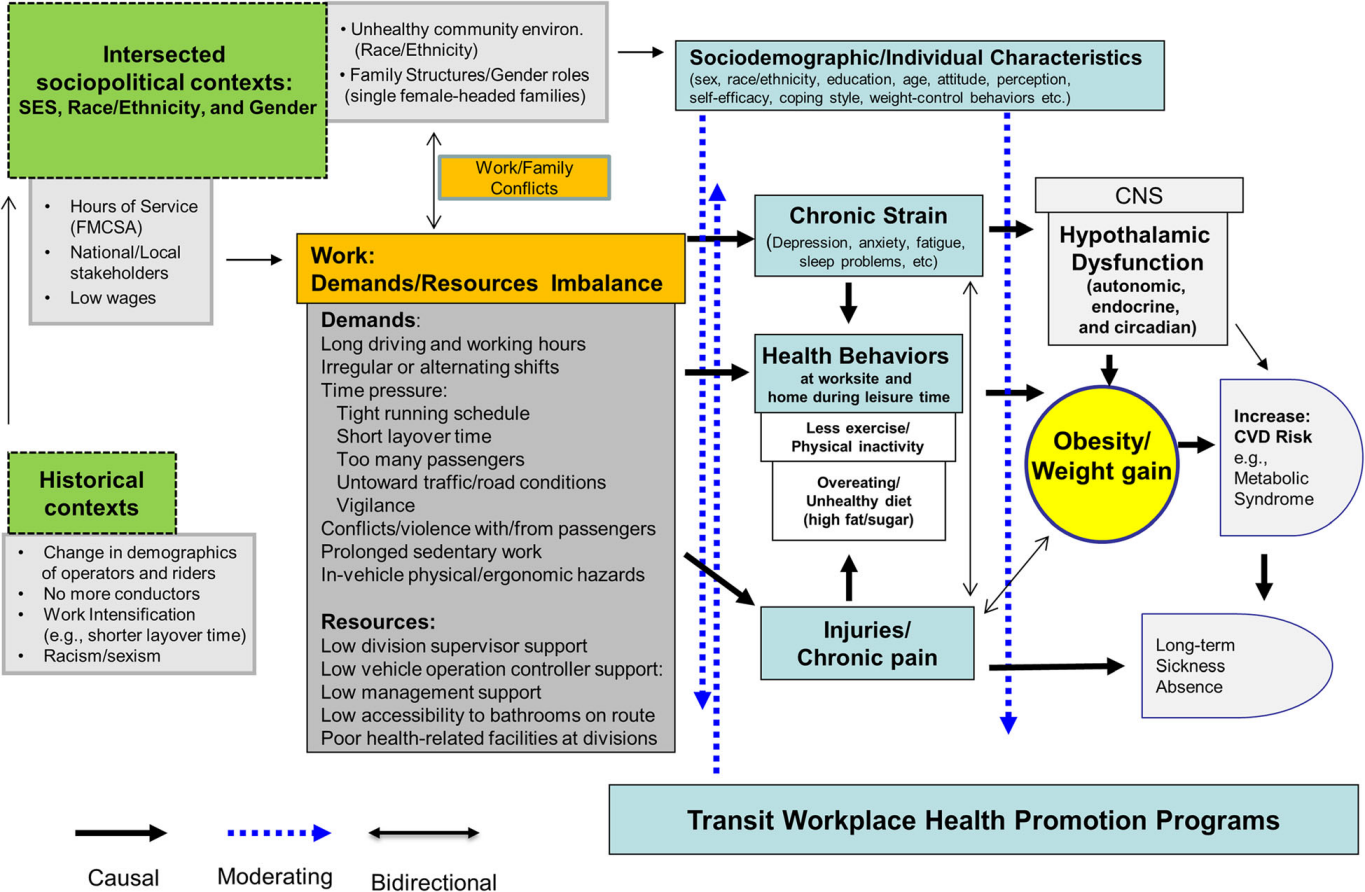
A theoretical framework on working conditions, chronic strain, health behaviors, obesity, and obesity disparity in diverse urban transit operators based on gender, race, and ethnicity. CNS: central nerve system. CVD: cardiovascular disease

The objective of this paper is to describe our newly developed socioecological framework for studying working conditions, chronic strain, health-related behaviors, weight gain/obesity, and obesity disparity among US transit operators (see Fig. 1). Compared to individual-based health behavior frameworks [28], socioecological frameworks on health and health behaviors [29–31] emphasize the influence of multi-level (e.g., micro-level, worksite; macro-level, community and society) environmental factors on individual’s and group’s health and health behaviors. Our framework is a synthesis of several different theories and disciplines: the theory of intersectionality (gender, and race/ethnicity), the resource-work load model (work stress), occupational ergonomics (work-related injuries and chronic musculoskeletal disorders), and worksite health promotion (for details, see below). This framework also incorporates results of our recently completed qualitative study that included five focus groups with 65 Los Angeles County Metropolitan Transit Authority (LACMTA) transit operators on work, health-related behaviors, and health (obesity) [32], as well as interviews and meetings with the representatives of the LACMTA management, the Sheet Metal Air Rail Transportation (SMART) local unions, and the SMART-MTA Trust Fund (a joint labor-management wellness program), and ride-along observations.

LACMTA employs over 4500 bus and 500 rail operators and provides bus and rail services to over 9 million Los Angeles county residents, who service over 500,000 riders per day. There are 18,500 stops on 189 lines in Metro Local and 22 Divisions spread throughout greater Los Angeles County. In 2006, the LACMTA began a wellness program as a joint labor-management program. The program includes a full-time wellness program coordinator and 1–2 bus or rail operators who work also as wellness ambassadors at each division. The ambassadors assist wellness activities at each division on “Wellness Wednesdays” and are paid from 9 am to 4 pm. They also participate in regular 1-h wellness meetings with the program coordinator every other Thursday.

## Development of a new socioecological framework for studying working conditions, chronic strain, health-related behaviors, weight gain/obesity, and obesity disparity in urban transit operators

Additional information on the role of occupational risk factors for obesity among transit operators is critical for designing more effective and sustainable obesity intervention programs for transit operators [33–37]. However, there has only been one cross-sectional epidemiological study [22] of occupational risk factors conducted in transit drivers and it examined only one occupational obesity risk factor: working more than 50 h/week was associated with higher BMI only in male transit operators. The study also found that long work hours were not associated with leisure-time exercise and consumption of fruits/vegetables in both male and female operators. But, in a small scale qualitative study [38], some Australian urban transit operators reported shift work and irregular driving times as barriers to their physical activity at work and during leisure time.

On the other hand, there is emerging evidence for the longitudinal associations between adverse working conditions and weight gain/obesity in non-transit working populations [39, 40]. The evidence is relatively strong for shift work [41–43] and long work hours [44–47] but as yet relatively weak for prolonged sedentary work [48–52], psychological job demands (time pressure and conflict demands) [53–56], job strain (a combination of low control and high psychological demands) [55, 57–59], and social support at work [55, 60]. No studies have examined the unique work schedule system of urban transit operators, nor the multiple psychosocial stressors leading to chronic strain in relation to adiposity [39]. Also, as noted above, the evidence for the association between long work hours and obesity in transit operators is still limited.

### Work schedule system of urban transit operators

Urban transit operators are among the few occupational groups in the US whose work hours are regulated by a nation-wide (federal) law. There is no nation-wide regulation limiting the maximum weekly work hours of the general working populations in the US. The mission of the US Federal Motor Carrier Safety Administration (FMCSA) within the Department of Transportation is to prevent commercial motor vehicle-related fatalities and injuries. According to the regulations on hours of service by the FMCSA, operators can drive up to 10 h/day and work up to 15 h/day. But they should have eight consecutive off-duty hours before driving again (https://www.fmcsa.dot.gov/regulations/hours-service/summary-hours-service-regulations). Operator work schedules vary by whether they are extra-board members (who are low-seniority full-time operators on weekly or daily changing work schedules and frequently covering absent operators) and on a “straight” (not unusually starting very early or late, sometimes alternating), split shift (divided into two work periods with a 2–3 h non-work period), or mixed shift [19, 61]. Urban transit agencies rely on overtime work of transit operators (particularly, extra-board operators) to deal with varying demands for bus and rail services by the time of day, the day of the week, and time of year [62]. Transit operators also have a strong incentive for overtime work because their hourly wage is low: as of 2015, the median hourly wage for US bus drivers was $14.88/h [10] and the top hourly wage for a “Tier 2” (hire after 1997) bus driver in the LACMTA was $23.94/h [32].

### Some adverse working conditions of transit operators may increase the risk for weight gain and obesity

Typical working conditions of transit operators are relevant to studies of obesity. The earlier two-person crew system (one driver and one conductor) for operating the bus has been largely replaced with a single-person (only one driver) system in major Western cities [12, 63]. Ethnic minority groups, particularly African Americans and Hispanic Americans, entered transit occupations in greater numbers after the 1970s with the closing of industrial manufacturing plants in Los Angeles urban areas [64]. Driving conditions have changed a great deal; for example, rest/recovery time of LACMTA transit operators at the end of each route has significantly decreased (e.g., 15–20 min in 1980s to 6–10 min in 2006) [64] and traffic conditions have worsened in many areas due to increased vehicles utilizing the streets. Worsening traffic conditions increase the risk of accidents and injuries to passengers and drivers. Driving requires heightened vigilance by drivers, leading to chronic physiological arousal (as manifested by higher blood pressures while driving) [65] in drivers compared to other occupations.

Gardell and his colleagues [57] proposed a work stress model (the resource-work load model) for predicting health risks among Swedish transit operators. The model is similar to the Karasek’s Demand-Control model [66]. However, the concept of work load in the resource-work load model is more specific for transit operators (e.g., lags behind the timetable, conflicts between timetable and safety, and conflicts between timetable and service) and the concept of resources is broader than the concept of control in the Karasek’s Demand-Control model as it includes several multi-level, different aspects of resources: authority on the job (how to handle problematic passenger contacts), technical (radio and alarms systems for information and security), social (coworker and supervisor support), and organizational resources (resources available through trade unions). According to their model, the greatest health risk among transit operators is expected in those who are exposed to the combined working conditions of low resources and high work load. For our theoretical framework (Fig. 1), as compared to the resource-work load model, we adopt broader concepts of job demands and job resources as risk factors for obesity in transit operators.

We hypothesize that high job demands and low job resources are the most important occupational adiposity risk factors independently and in combination with each other (e.g., the resource-work load model) [61] among transit operators (Fig. 1). High job demands include long driving and work hours (e.g., >70 h/week and >12 h/day), irregular shifts (e.g., extra-board operators and alternating between very early and late shifts), conflicts with passengers and verbal and physical violence from passengers, prolonged sedentary work (e.g., > 85% of their work hours), time pressure, in-vehicle ergonomic hazards, and work and family conflict. Time pressure is defined here as efforts to maintain the running schedules while meeting possible competing demands of driving safely and serving the public [61, 67], including short layover time (a break time for operators’ rest, eating, and access to the restroom at the end of each route). It could be operationalized as a multidimensional measure of short intervals between stops, high passenger load, high traffic load, constant threat avoidance vigilance (TAV), and short layover time. TAV is a concept advanced by Dr. Karen Belkic and her colleagues to describe work settings in which workers must maintain constant vigilance to avoid catastrophe – exactly the situation faced by bus operators in urban areas [65]. Low job resources include low division supervisor/vehicle operation controller/management support, low accessibility to bathrooms (toilets) on route, and poor health-related facilities at divisions. All of these adverse working conditions were identified as stressful occupational factors or barriers to health-related behaviors in our pilot project with 65 operators [32], which is similar to qualitative work stress studies in British and Australian transit operators [63, 68].

We identified a substantial variation in each of the aforementioned occupational risk factors for obesity among LACMTA transit operators in our recent pilot study. For example, about 20% of LACMTA operators in our pilot project worked >12 h/day and 14% worked >70 h/week. We hypothesize that adiposity risk will be the highest in the long driving and working hour operators, particular in dense traffic areas imposing high time pressure and requiring greater TAV. Long driving and working hours were associated with obesity in recent truck driver studies [69, 70]. The Framingham 10-year coronary heart disease risk scores were significantly greater in the bus drivers who worked long hours (>12 h/day) than in those who worked regular work hours (8 h/day) in a Korean bus driver study [21]. We also hypothesize that adiposity risk will be higher in the operators doing irregular work shifts (vs. fixed shifts) and who are exposed to high time pressure (vs. low time pressure). While there has been an overall decrease in the layover time of transit operators since the 1980s [64], layover time varies significantly among individual operators [71, 72].

There is also a non-trivial variation in sedentary work time among transit operators: > 96 min during an 8-h work [73]. Sedentary work time of transit operators can accumulate during driving (driving time plus sitting time during layover time) and non-driving work hours [72] including waiting time for assignment at divisions [74]. We hypothesize that adiposity risk will be greater in the operators doing longer sedentary work (>85% of their work hours vs. ≤ 85%) [73]. In addition, conflicts with passengers on the bus particular fare and verbal abuse and physical assaults are not rare, particularly in the routes that go through low-income communities. Poor in-vehicle ergonomic hazards (e.g., vibration and seat) [17] may increase adiposity risk among operators through work-related injuries/chronic pain (for details, see below). Also, emotional and information support from division supervisors and vehicle operation controllers are very important for operators’ work performance and mental health [32]. Several LACMTA female operators in our pilot project reported more work and family conflict (child care and contact with children) when they worked longer particularly on irregular shifts.

### Etiological mechanisms for research on adverse working conditions and adiposity risk in diverse urban transit operators based on gender, race, and ethnicity

We propose five important etiological mechanisms (Fig. 1) by which the aforementioned adverse working conditions can increase adiposity risk in operators. These five mechanisms are not mutually exclusive and multiple factors are likely to be active at the same time. The first three mechanisms are consistent with contemporary theoretical frameworks on work and obesity suggested previously by our group [26] and Pandalai et al [25]. But we further explicate the first mechanism (see below) and the last two mechanisms are newly presented here.

First, the aforementioned adverse working conditions of high job demands (except for prolonged sedentary work), low resources, or their combination may function as stressors to induce chronic strain (e.g., depression, anxiety, fatigue, and sleep problems) resulting in dysfunction of the hypothalamus [75–78] that could shift energy balance towards the positive via alterations of the autonomic nervous system, endocrine systems, and circadian rhythms in relation to lipid metabolism [75, 79–87]. For example, long and irregular work hours can result in short or poor sleep hours that are associated with BMI among drivers [7, 69].

Here we describe the first mechanism with a focus on lipid metabolism in detail from the neuropsychological, endrocrinological, and chronobiogical perspectives. The dysfunction of the hypothalamus includes both hyper- or hypo-activations of the hypothalamus. Cortisol is secreted by the activation of the hypothalamus-pituitary-adrenal cortex axis. The level of blood cortisol has a unique diurnal pattern in humans: a rapid increase just after wakening, the peak (acrophase) about 30 min after awaking, and the bottom (nadir) before bedtime. Depressed individuals had lower levels of cortisol than non-depressed individuals in the morning. However, in the afternoon, depressed individuals had higher levels of cortisol, but lower reactivity to a stressor, compared to non-depressed individuals [78]. It is also well-known that patients with chronic use of corticosteroids become centrally obese and have an increased appetite [77]. High levels of cortisol may induce a resistance to leptin that is produced by adipocytes and increases satiety in the hypothalamus [77]. In rats, chronic exposure to high levels of corticosterone increased visceral adiposity, inhibited the lipolysis of triglycerides (stored fat) into fatty acids in mature adipocytes, and increased the differentiation of premature adipocytes into mature adipocytes (adipogenesis) [88].

Norepinephrine and epinephrine are released by the activation of the sympathetic nerve system that is under the control and coordination of the hypothalamus [87, 89]. The hormones increase lipolysis through β-(particularly its subtype, β_3_) adrenergic receptors in adipocytes [79, 90–95]. It is known that chronic use of β-adrenergic receptor blockers as antihypertensive medications increase the risk for weight gain [96]. Under chronic stress, the adrenergically mediated lipolysis in adipocytes may be inhibited or impaired by high levels of cortisol. Cortisol represses β_3_- adrenergic receptor gene expression (transcription) in white and brown adipocytes in mice [90]. Interestingly, the adrenergically stimulated lipolysis in white adipocytes was blunted (reduced) in obese humans [79]. High cortisol levels may also decrease the thermogenesis of brown adipocytes [92, 93, 97] by repressing the β_3_ adrenergic receptor gene transcription in brown adipocytes, which remains to be tested in the future.

Disturbance of biological circadian rhythmicity can increase the risk for obesity given the reciprocal interrelationship between metabolism and circadian clocks [87, 98]. The central master circadian clock is located at the suprachiasmatic nucleus of the hypothalamus, while the peripheral clocks are found in liver, pancreas, muscle, and adipose tissue. Several hormones and enzymes that are involved in lipid metabolism are under the control of the biological clocks and have their own circadian rhythms. For example, leptin has its peak level just after midnight in humans [99]. Sleep restriction in healthy men changed the leptin circadian rhythmicity (earlier peak time and dampened rhythmicity) and reduced the 24-h mean level of blood leptin [100]. In nocturnal mice, mice fed a high-fat diet during the day phase were significantly heavier than mice fed the isocaloric high-fat diet during the night phase [101]. Lipoprotein lipase in adipose tissue takes up fatty acids in blood for use and storage in adipose tissue. The gene expression for the lipase in adipose tissue after a normal or high-fat diet significantly increased in mice without a biological clock-related nuclear receptor (REV-ERBα), while it did not change in mice with the receptor [102].

On the other hand, the aforementioned stressors may also increase the risk for elevated heart rate, hypertension, hyperlipidemia, hyperglycemia, arterial inflammation, and arrhythmia among transit operators through chronic psychological and physiological arousal, including activation of the hypothalamus and amygdala [65, 103–106] contributing to the development of the metabolic syndrome, atherosclerosis, and CVD. The increased CVD risk by the stressors may be a major contributor to high long-term sickness absence and workers’ compensation claims in transit operators [10, 18].

Second, the aforementioned adverse working conditions of high job demands (except for prolonged sedentary work), low resources, or their combination may cause obesity via changes in health behaviors (exercise and eating behaviors at worksite and home during leisure-time) [39, 107–115]. Some LACMTA operators in our pilot project reported that their work schedule (e.g., long work hours/overtime, irregular shift, and time pressure) forces them to eat fast and also they tend to eat a lot of “comfort foods” when stressed at work.

Third, low work-related physical activity (e.g., prolonged sedentary work), given a constant level of energy consumption, could lead to a positive energy imbalance state [116–118] contributing to weight gain.

Fourth, adverse working conditions of high job demands, low resources, or their combination may increase the risk for weight gain/obesity via work-related injuries [16, 17] or chronic severe pain on muscle/joints [27] that in turn affect health behaviors (e.g., exercise). In-vehicle ergonomic hazards, driving hours, and low supervisor support were associated with low back injury in transit operators [16, 17, 119]. Injury is one of the main obstacles to maintaining or adopting exercise in blue-collar workers [23, 120]. Also, there may be a bidirectional relationship between severe chronic pain and sleep disturbance [121–123]: severe chronic pain may negatively affect sleep quality and mental health, which in turn reduces pain tolerance, and vice versa. On the other hand, adiposity can also increase the risk for injuries/chronic pain [124, 125].

Fifth, minority women operators may be exposed to more adverse occupational and non-occupational (community and family) conditions than male and other female operators. The theory of intersectionality [126–128] highlights the possible synergistic detrimental health effects of the combination of socioeconomic status, gender, and race/ethnicity. Crenshaw [126] developed the theory of intersectionality as a critique of a single-axis framework in antidiscrimination law, feminist theory, and antiracist politics in the 1980s: for example, in race discrimination discourses, a single axis (sex-based or class-based) approach is not appropriate for addressing the experiences of Black women who were multiply-burdened (i.e., low social class, women, and Black). Recently, several public health researchers have started to pay attention to the theory of intersectionality for research in multiple historically oppressed populations [127, 128]. We think that the theory of intersectionality serves as a theoretical basis for examining the intersected relationships of low social class, being a woman, and a member of a racial/ethnic minority in understanding obesity disparities between minority women operators, and male and other female operators.

Female operators were more stressed from irritated and aggressive passengers and congested traffic conditions than male operators [129]. Also, several LACMTA female operators in our pilot project reported more work and family conflict (child care and contact with children) when they worked longer particularly on irregular shifts. Furthermore, long work hours, when combined with long domestic familial duties, may reduce leisure-time exercise of female operators [130]. All these factors are more likely to be experienced by minority women operators given the more common single female-headed families among African Americans (43% vs. 13% among whites) [131], less accessible residential health promoting environments (e.g., parks and supermarkets providing healthy foods) in minority communities [132, 133], and also racism and sexism in our society [134, 135]. Additionally, work stress coping behaviors and tolerance of heavier body weight may differ by gender and race/ethnicity [136–141], which further supports the possible differential relationships between working conditions, health behaviors, and adiposity among transit operators.

### Main study domains to be measured for empirically testing our theoretical framework

In order to facilitate future worksite obesity studies among transit operators based on our new theoretical framework, we present a list of key domains that should be assessed with available instruments in the literature in the future studies.

- *Working conditions*: detailed work schedules, sedentary work time, time pressure (short intervals between stops, high passenger load, high traffic load, constant threat avoidance vigilance, and short layover time) [61, 66], coworker and supervisor support [14, 116, 142], conflicts with passengers [64, 143], physical demands [142, 144], worksite food/physical activity environment [145], worksite discrimination [146–148], and in-vehicle ergonomic hazards (eight-item scale about the seat, vibration, and heat/cold/draft) [17]
- *Health behaviors at work and during leisure time*: eating behaviors and exercise at worksite and at home [92, 108, 149, 150], including stress-induced overeating, weight control behaviors [151], and impaired exercise/physical activity due to work-related injuries/chronic severe pain in the past year [152]
- *Health conditions*: weight perception (body image), work-related injuries (resulting in > 2 weeks of sickness absence in the past year) [153, 154], chronic severe pain on muscle/joints in the past year (lasting > 30 days) [27, 119, 152], mental health (depression, exhaustion, sleep quality and hours. etc.), and chronic diseases (hypertension, hyperlipidemia, and diabetes)
- *Community and family variables*: neighborhood and community health promotion facilities [155], work-family conflicts [156], and family structure variables including household head, number of young children, and household work hours [129]
- S*ociodemographic variables*: age, gender, race/ethnicity, and individual and household income.
- *Adiposity (weight, height, and waist circumference)*. For the calculation of BMI, measured weight and height. Also, waist circumference needs to be measured considering its stronger CVD prediction than BMI [157–159] and a possible overestimation of adiposity by BMI [160–162] in African Americans. Obesity will be defined with the current standard cut-points for BMI (>30 kg/m^2^) and WC (>40 in. for men and >35 in. for women) [163]

## Conclusions

In this paper, we proposed a socioecological framework for research on working conditions, chronic strain, health-related behaviors, weight gain/obesity, and obesity disparity in urban transit operators with diverse gender and race/ethnicity backgrounds (Fig. 1). Compared to our previous framework [26], we have further explicated some physiological mechanisms (e.g., dysfunction of hypothalamus) by which adverse working conditions increase the risk for obesity from the neuropsychological, endrocrinological, and chronobiogical perspectives. This new framework also provides further insight, when compared to our previous and other contemporary theoretical frameworks on work and obesity/health [24–26], in explaining the intersected obesity disparity between minority women transit operators and male and other female transit operators in the US. Our new framework, albeit exploratory, is unique in that it embraces and highlights injuries/chronic severe pain as a possible pathway from adverse working condition to weight gain/obesity among transit operators who are at high risk for non-fatal occupational injuries and chronic back and neck pain.

### Comparisons with our previous framework for research on work and obesity in professional firefighters

Our new framework for research on work and obesity in transit operators described in this paper is a self-critical and innovative extension of our previous framework for research on work and obesity in professional firefighters [26]. Our previous theoretical framework was developed for research on work and obesity in professional firefighters who are mostly male and mostly white in a suburban county of Southern California. Our previous theoretical framework has successfully guided a research project (called the FORWARD study) that has shed new light on the inter-relations between working conditions, health-related behaviors, obesity, and CVD risk factors among professional firefighters [116, 164, 165].

Nonetheless, our previous theoretical framework is limited in application to research on work and obesity in urban transit operators due to the following reasons. First, the work/rest schedule of transit operators is largely regulated by the FMCSA federal (national) regulation, while the work shift schedule of professional firefighters is determined through collective bargaining between the local fire department and local firefighter union. This observation led us to consider theoretically a broader socioecological perspective, including both the macro-level (national polices and regulations) and micro-level (worksite) contexts which is more consistent with the aforementioned multi-level socioecological health promotion models [29–31]. Our previous theoretical framework was exclusively centered on local worksite contexts and lacked macro-level (national or societal) contexts. Second, bus drivers, as contrasted with firefighters and workers in other occupations, have unique working conditions, that of driving a bus, that place them in situations requiring continuous vigilance contributing to chronic arousal. Third, there is a substantial obesity disparity between minority women operators and male and other female operators. Our previous theoretical framework developed for research in predominantly white male professional firefighters could not appropriately explain obesity disparity within a single occupational group. This dilemma could be resolved in our new theoretical framework by employing the theory of intersectionality with a focus on the potentially differential experiences of minority women operators not only at the workplace, but also at the residential (community) and society levels. Fourth, obesity and work-related injuries/chronic severe pain are both prevalent among some blue collar workers, including transit operators and firefighters. However, as mentioned before, work-related injuries and severe chronic pain were not considered in our previous theoretical framework in professional firefighters. In contrast, our new theoretical framework suggests a possible causal relationship between adverse working conditions, work-related injuries/chronic pain, and weight gain/obesity in working populations based on an extensive literature review on occupational ergonomics (impact of psychosocial and physical working conditions on work-related injuries and chronic pain), worksite health promotion (health behaviors), and sleep/stress. On the other hand, the work stress model by Gardell and his colleagues [61] was a very helpful platform for us to specify several job demands and resources as occupational risk factors for obesity among urban transit operators.

### Implications for future worksite obesity interventions in urban transit operators

Our new framework has some important implications for future intervention studies for improving the cardiovascular health (e.g., obesity, hypertension, and metabolic syndrome) of urban transit operators. As mentioned before, the previous two 18-month intervention studies [5, 23] were not successful in reducing or preventing obesity/BMI increase among transit operators: healthy vending machine choices at division buildings [5] and typical worksite health promotion programs based on health education and individual/group activities [23] were not efficacious. Our new framework points to adverse working conditions as potential “high impact leverage points” [166, 167] for obesity interventions and other CVD risk factor interventions (e.g., high blood pressure) among urban transit operators. Previous worksite environmental interventions for improving weight status have been confined to physical environmental changes such as onsite food availability and onsite exercise facilities [35].

Several successful European intervention studies targeted adverse working conditions of transit operators (bus only lanes, reducing curves on the route, improving information service for the public, installing new driver’s seats, work-hour reduction, improving communication, and creating semi-autonomous teams of drivers) [168–172]. The interventions successfully resulted in lowered perceived workload, psychosocial stressors and, in some cases lower CVD risk factors (resting systolic blood pressure and heart rate at work), sickness absenteeism, job dissatisfaction, psychological distress, and body pain. Although none of these successful interventions have examined weight status or obesity as a key intervention outcome, they support that adverse working conditions are changeable for improving the health of transit operators.

Our new framework also clearly indicates that in order to improve the cardiovascular health of transit operators, transit stakeholders in addition to changes in job demands and job resource should also make efforts to address macro-level (national and community) determinants of adverse working conditions and health behaviors of transit operators. For example, the FMCSA hours of service (work and rest) regulation for commercial vehicle drivers has been revised, discussed, and sometimes suspended. A new regulation for reducing fatigue among truck drivers was introduced in 2013. The 34-h restart rule: for a restart, drivers should have at least 34 h off-duty time after their 60- or 70-h work schedule and the 34-h off-duty time should include two periods of 1 am to 5 am. However, it has been suspended until the end of an on-going safety and health research project on the new regulation (https://www.fmcsa.dot.gov/regulations/hours-service/summary-hours-service-regulations). If necessary, a similar regulation may be implemented for transit operators in the future. Transit stakeholders can work together with minority community stakeholders (e.g., community and political leaders, local businesses and organizations, health care providers, and local academic researchers) to create healthy food or exercise environments in minority communities [173–175].

Our hypotheses highlighted above in the new theoretical framework are that some adverse working conditions (long driving and working hours, irregular or alternative shifts, time pressure including short layover time and constant threat avoidance vigilance, conflict with passengers, violence from passengers, prolonged sedentary work, in-vehicle physical/ergonomic hazards, low supervisor and management support, work-and-family conflict, low accessibility to bathrooms on route, and poor health-related facilities at division buildings), largely characterized as a combination of high demands and low resources, will all and in part increase the risk for weight gain/obesity among transit operators directly through chronic strain and dysfunction of hypothalamic regulation, and indirectly through health-related behaviors at the worksite and during leisure-time as well as through injuries/chronic severe pain. In addition, adiposity risk will be greater in minority women operators due to their greater exposure to adverse occupational and non-occupational conditions.

Our etiological framework and hypotheses on adverse working conditions and obesity risk among transit operators remain to be tested and refined through future observational, intervention, and field biological monitoring (e.g., salivary cortisol and ambulatory heart rate, heart rate variability, and blood pressure) studies. Nonetheless, we think that our newly developed framework is a good theoretical guide and will significantly facilitate future transit worksite obesity studies by a joint research team of interdisciplinary university researchers and transit stakeholders. Also, the framework, if confirmed in future studies, will contribute to understanding the complex and important roles of adverse working conditions in the etiology of weight gain/obesity and obesity disparity among transit operators and other working populations.

## Abbreviation

BMI: Body mass index
CVD: Cardiovascular disease
FMCSA: Federal Motor Carrier Safety Administration
FORWARD: Firefighter Obesity Research: Workplace Assessment to Reduce Disease
LACMTA: Los Angeles County Metropolitan Transit Authority
REV-ERBα (known as NR1D1): Nuclear Receptor Subfamily 1, Group D, Member 1
SMART: Sheet Metal Air Rail Transportation
TAV: Threat avoidance vigilance
US: United States
